# Nested Case–Control Study of Autoimmune Disease in an Asbestos-Exposed Population

**DOI:** 10.1289/ehp.9203

**Published:** 2006-05-30

**Authors:** Curtis W. Noonan, Jean C. Pfau, Theodore C. Larson, Michael R. Spence

**Affiliations:** 1 Center for Environmental Health Sciences, University of Montana, Missoula, Montana, USA; 2 Agency for Toxic Substances and Disease Registry, Centers for Disease Control and Prevention, Atlanta, Georgia, USA; 3 Montana Department of Health and Human Services, Helena, Montana, USA

**Keywords:** asbestos, autoimmune, Libby, lupus, rheumatoid arthritis, scleroderma, vermiculite

## Abstract

**Objective:**

To explore the potential association between asbestos exposure and risk of autoimmune disease, we conducted a case–control study among a cohort of 7,307 current and former residents of Libby, Montana, a community with historical occupational and environmental exposure to asbestos-contaminated vermiculite.

**Methods:**

Cases were defined as those who reported having one of three systemic autoimmune diseases (SAIDs): systemic lupus erythematosus, scleroderma, or rheumatoid arthritis (RA). Controls were randomly selected at a 3:1 ratio from among the remaining 6,813 screening participants using frequency-matched age and sex groupings.

**Results:**

The odds ratios (ORs) and 95% confidence intervals (CIs) for SAIDs among those ≥ 65 years of age who had worked for the vermiculite mining company were 2.14 (95% CI, 0.90–5.10) for all SAIDs and 3.23 (95% CI, 1.31–7.96) for RA. In this age group, exposure to asbestos while in the military was also an independent risk factor, resulting in a tripling in risk. Other measures of occupational exposure to vermiculite indicated 54% and 65% increased risk for SAIDs and RA, respectively. Those who had reported frequent contact with vermiculite through various exposure pathways also demonstrated elevated risk for SAIDs and RA. We found increasing risk estimates for SAIDs with increasing numbers of reported vermiculite exposure pathways (*p* < 0.001).

**Conclusion:**

These preliminary findings support the hypothesis that asbestos exposure is associated with autoimmune disease. Refined measurements of asbestos exposure and SAID status among this cohort will help to further clarify the relationship between these variables.

An association between occupational exposures of inhaled particulates and autoimmunity was postulated as early as 1914, when Bramwell (1914) reported increased frequency of diffuse scleroderma (SSc) in stone masons. Although genetic factors undoubtedly exist that affect the development of systemic autoimmune diseases (SAIDs) in certain individuals, the concordance of SAIDs among identical twins is only 25–40%, suggesting that environmental factors play a substantial role (Powell et al. 1999). Indeed, several environmental agents are implicated in triggering or accelerating SAIDs, including mercury, iodine, vinyl chloride, certain pharmaceuticals, and crystalline silica. However, much more research is needed to determine the mechanisms and epidemiology linking exposures to development of SAIDs. There is considerable epidemiologic evidence supporting the hypothesis that occupational silica exposure is associated with a variety of SAIDs, including SSc, rheumatoid arthritis (RA), systemic lupus erythematosus (SLE), glomerulonephritis, and small vessel vasculitis (Koeger et al. 1995; Parks et al. 1999, 2002; Powell et al. 1999; Steenland and Goldsmith 1995). Research regarding asbestos exposure and SAIDs has been much more limited.

Asbestos-related lung disease continues to be a serious and significant problem worldwide despite increasing awareness of health hazards of asbestos inhalation. Asbestos exposure is associated with various lung conditions, including fibrosis, pleural plaques, and cancer. Although the exact mechanisms leading to the progression of these conditions have not been fully explained, there is evidence that some of the lung abnormalities seen with both asbestos and silica exposures are immunologically mediated (Hamilton et al. 1996; Holian et al. 1997; Perkins et al. 1993). Nevertheless, it is unclear how these innate immune responses might translate to specific humoral responses. Increased serum immunoglobulins (Ig), positive antinuclear antibody (ANA) tests, and immune complexes have been reported in small cohorts of individuals exposed to asbestos (Lange 1980; Nigam et al. 1993; Pfau et al. 2005; Zerva et al. 1989), but no comprehensive study has been undertaken to assess the association between asbestos exposure and autoimmune disease.

Our major objective, therefore, is to establish whether such an association exists, and the community of Libby, Montana, provides a unique opportunity to investigate this question. Individuals in this population experienced significant exposures that occurred as a result of asbestos-contaminated vermiculite mining near the community. From the early 1920s to 1990, the world’s largest vermiculite deposits, located near Libby, were mined and processed. Vermiculite is a silicate mineral with unique properties and numerous commercial applications (Lockey 1984). The fibrous minerals contaminating Libby vermiculite have been characterized as both regulated asbestos fibers (e.g., tremolite and other amphibole forms) and unregulated fibers (e.g., winchite and richterite) (Meeker et al. 2003). The various mining, transportation, and processing activities as well as the personal and commercial use of vermiculite in the community have led to widespread environmental exposures in the Libby area with this asbestos-contaminated vermiculite. Potential asbestos exposures in this community have been documented not only in the miners but also in their family members as well as anyone who used the vermiculite or played near the mine tailings (Dixon et al. 1985). A mortality study in this community found more than 40-fold increases in standardized mortality ratios for asbestosis, and elevated mortality also was observed for malignant neoplasm of respiratory and intrathoracic organs (Horton et al., in press).

Recently, the Agency for Toxic Substances and Disease Registry (ATSDR) conducted an extensive screening program of > 7,300 individuals from this community (Peipins et al. 2003). The initial results of this screening program identified various routes of exposure in the community and how those routes of exposure were associated with abnormalities on chest radiographs (Peipins et al. 2003). In addition, when the ATSDR performed its screening in Libby during 2000–2001, 494 (6.7%) participants indicated that they had been diagnosed with SLE, SSc, or RA (Noonan et al. 2005). By comparison, a prevalence of < 1% for these three conditions combined would be expected based on pooled estimates from 43 prevalence studies (Jacobson et al. 1997). In the present study, we take these data a step further by exploring the association of these systemic autoimmune conditions with various parameters of asbestos and/or vermiculite exposure using a nested case–control approach.

## Materials and Methods

All human subjects provided informed consent for this study under a protocol approved by the institutional review board for the Centers for Disease Control and Prevention. The details of the ATSDR screening program are described elsewhere (Peipins et al. 2003). Briefly, individuals were eligible for the screening program if they had resided, worked, attended school, or participated in other activities in the Libby area for at least 6 months before 31 December 1990. All screening participants who were ≥ 18 years of age and not pregnant (*n* = 6,668) were offered chest radiographs. Two independent B readers evaluated the radiographs for each subject for pleural and parenchymal abnormalities. If these two readers disagreed regarding the presence of pneumoconiosis for a subject, a third reader was used to adjudicate the difference. Participants were classified as “positive” for pleural or parenchymal abnormalities if at least two of three B readers observed this type of abnormality on chest radiographs. Participants also received spirometry testing and were considered to have abnormal findings if they had a forced vital capacity (FVC) < 80% predicted and a ratio of 1 sec forced expiratory volume (FEV_1_) to FVC that is ≥ 70% predicted. Data on exposure to asbestos-contaminated vermiculite were based on occupational, residential, and recreational histories collected during in-person interviews. Demographic variables and data on other potential covariates were also collected by in-person interview.

**Figure 1:**
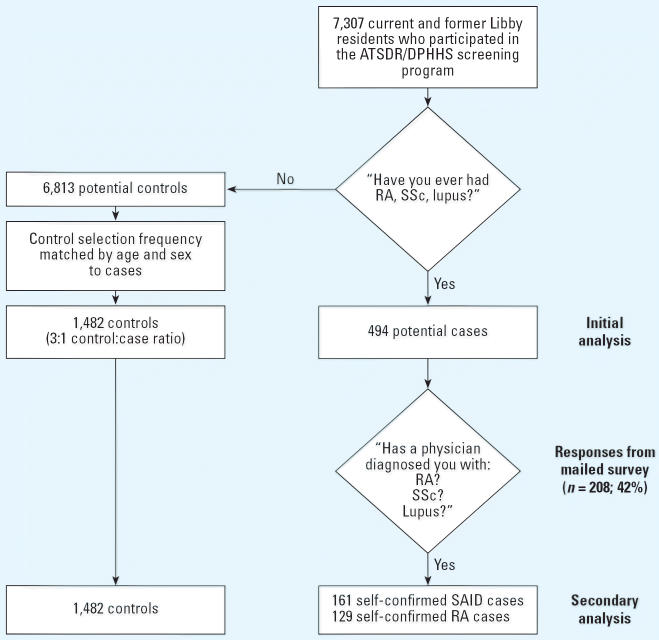
Case and control selection from initial screening population. DPHHS, Montana Department of Public Health and Human Services. Lupus refers to SLE.

This study was conducted in two phases. The initial characterization of cases (*n* = 494) with SAIDs were those participants who, during the 2000–2001 ATSDR screening program, responded affirmatively to the question “Have you ever had rheumatoid arthritis, scleroderma, or lupus?” Potential controls were those screening participants who answered negatively to this question. Controls were randomly selected from within strata of sex and 10-year age groups at a 3:1 control-to-case ratio (*n* = 1,482) (Figure 1).

The initial screening question on SAIDs was collected only to identify screening participants with health conditions that could have an impact on pulmonary function or fibrosis. The second phase of this study involved a mailed questionnaire to confirm the original self-reports of SAIDs and to identify which of the three conditions the potential cases were reporting. The follow-up survey was mailed to all 494 potential SAID cases for whom current addresses were available. The follow-up survey queried potential cases on whether or not they still considered themselves to have one of the three indicated SAIDs, which SAID(s) they had, whether or not their SAIDs were diagnosed by a physician, and whether or not they were taking medication or other treatment for their condition. For those reporting RA, additional questions were asked to confirm the type of arthritis on which they were reporting (i.e., RA, osteoarthritis, or general arthritis). This follow-up survey was approved by the University of Montana investigational review board.

Analyses were conducted using unconditional logistic regression using SAS (version 9; SAS Institute Inc., Cary, NC). The presence or absence of SAIDs or a specific autoimmune disease in the postmailing analysis was used as the dependent variable. The various pathways of exposure to vermiculite and/or asbestos were considered as the main independent variables of interest. Test for trend with increasing numbers of exposure pathways was assessed using the Cochran-Armitage test. Potential confounders included indications of restrictive spirometry and the presence of pleural or parenchymal abnormalities. These pulmonary features were the main outcomes of the ATSDR screening program and could be independently associated with both asbestos exposure and biomarkers of autoimmunity (Pfau et al. 2005). For the final unconditional logistic regression models, all vermiculite/asbestos exposure pathways and other potential risk factors were considered. Criteria for inclusion in the final model included statistical significance of the explanatory variable (*p* < 0.10), the presence of a confounding effect on other variables, and the fit of the model.

## Results

The distribution of SAID subjects for selected characteristics are presented in Table 1. Among the 494 subjects responding positively to the original SAID screening question, 287 (58%) were women. More than 75% of SAID subjects lived in Libby for at least 15 years. Follow-up mailed questionnaires were sent to all 494 subjects who were classified as having SAIDs in the initial analysis. Of these, 208 (43%) participants responded. Among those responding, 161 participants confirmed that they had a physician-diagnosed SAID. The proportional distribution of those reporting physician-diagnosed SAIDs was similar to the original 494 who reported SAIDs with regard to sex, age, smoking history, and years lived in Libby (Table 1). Among those reporting physician-diagnosed SAIDs, 129 participants indicated that they had physician-diagnosed RA, 70% of whom took medication for RA. Thirty participants indicated that they had physician-diagnosed SLE, 63% of whom took medication for SLE. Another four participants indicated that they had SSc, and two of those took medication for SSc.

Considering the initial case group (*n* = 494), the distribution of years of residence was not different for cases and controls (χ^2^ = 0.57, *p* < 0.90). Current and former smokers were more common among SAID subjects [odds ratio (OR) = 1.72; 95% confidence interval (CI), 1.22–2.44]. SAIDs also were associated with parenchymal abnormalities and with restrictive spirometry but not strongly with pleural abnormalities (Table 2). These associations remained consistent when evaluating those reporting physician-diagnosed SAIDs (*n* = 161) (Table 2). Both restrictive spirometry and parenchymal abnormalities as well as current or former smoking were included in the unconditional logistic regression models for vermiculite/asbestos exposure and risk of SAIDs.

The adjusted ORs for various potential exposure pathways to vermiculite and asbestos and risk of SAIDs are presented in Table 3. Previous occupation with the mining company yielded no overall increased risk for SAIDs (OR = 1.03; 95% CI, 0.69–1.58). However, we observed elevated ORs for previous occupation with the mining company among those ≥ 65 years of age at the time of the screening, particularly for RA. Among other occupational vermiculite exposures, “dust or vermiculite exposure at jobs other than W.R. Grace/Zonolite” was the most consistently associated with reporting of any SAID or RA specifically. Among other occupations with potential exposure to asbestos, only those reporting asbestos exposure in the military were at increased risk for SAIDs and RA, yielding ORs of 1.70 and 2.11, respectively. Several reported activities or experiences were associated with increased risk of any SAID or RA specifically. Considering all exposure pathways collectively, increasing opportunities for vermiculite and/or asbestos exposure yielded increasing risk estimates for SAIDs and RA (Table 4). Although the number of SLE cases was too small (*n* = 30) to evaluate for trend as in Table 4, we did observe an elevated risk for SLE among those with more than three pathways of exposure versus those with three or fewer pathways of exposure (OR = 4.45; 95% CI, 1.24–16.00). We did not observe sufficient numbers of SSc cases to evaluate this disease by asbestos exposure pathways.

Table 5 presents final unconditional logistic regression models in which all exposure pathways and other potential risk factors were considered for their contribution to the risk of SAIDs and RA. Because older participants had differing occupational risk factors, we constructed models stratified by age. For participants < 65 years of age, dust or vermiculite exposure at jobs other than W.R. Grace/Zonolite was the only occupational exposure that remained in the models for both SAIDs and RA. For participants ≥ 65 years of age, asbestos exposure in the military yielded substantially elevated risk estimates for both SAIDs and RA. For this age group, working at W.R. Grace/Zonolite also resulted in a 3-fold greater risk for RA. Elevated ORs were also observed for several nonoccupational exposures to vermiculite (Table 5).

## Discussion

Although there is considerable epidemiologic evidence supporting the hypothesis that occupational silica exposure is associated with a variety of SAIDs, research regarding asbestos exposure and SAIDs has been much more limited. The preliminary findings presented here support the hypothesis that asbestos exposure is associated with the presence of autoimmune disease. We found increased risk for SAIDs among those reporting occupational and environmental or recreational exposures to vermiculite and/or asbestos. Increased risk estimates were found for increasing numbers of reported exposure pathways. The risk estimates by exposure pathway remained elevated and in some cases increased after restricting the cases to those who responded to a follow-up survey and confirmed that they had a physician-diagnosed SAID or RA specifically. We recognize the limitation of this approach of combining exposure pathways of unequal intensity and duration, but it also provides an analysis that parallels the previously observed findings of asbestos exposure and lung abnormalities in this population (Peipins et al. 2003). The multivariable analysis identified specific exposure pathways that were independently associated with risk of SAIDs, including older subjects who had worked for the mining company. Among older participants, we also observed increased risk estimates for SAIDs and RA among those reporting exposure to asbestos in the military. The risk for military asbestos exposure was independent of the elevated risk for previous work at W.R. Grace/Zonolite and other Libby exposure pathways. These findings suggested that asbestos exposure in general rather than Libby vermiculite exposure in particular could be relevant to SAID etiology.

These findings were consistent with a recent immunologic study we undertook among a small group of volunteers from this same Libby community (Pfau et al. 2005). The results of that study supported the hypothesis that increased frequency of positive ANA would be found in the Libby group compared to an age- and sex-matched unexposed population in Missoula, Montana. Among the Libby volunteers, the titers of the positive ANAs were positively correlated with the length of the individual’s estimated asbestos exposure. Previous studies have measured several immune parameters in populations exposed to asbestos. Investigators in India demonstrated increased IgG, IgA, and positive ANAs in asbestos-exposed individuals, compared to controls, even in the absence of apparent lung disease (Nigam et al. 1993). This finding suggested that immune alterations may precede the onset of asbestos-related disease. A high frequency of positive ANAs was also found in a Japanese group of asbestos plant workers (Tamura et al. 1993). Interestingly, a 3-year follow-up study of the Japanese group showed significant correlation of positive ANAs with progression of disease, leading to additional diagnoses of asbestosis in a previously healthy group (Tamura et al. 1996). A study of Greek residents exposed to asbestos-contaminated whitewash showed a correlation of immune abnormalities particularly in individuals with pleural plaques (Zerva et al. 1989), whereas the Japanese study showed a greater effect in individuals with diffuse fibrosis but not pleural plaques. In addition, a Polish study reported increased ANA frequency particularly in individuals exhibiting lung function deficits (Lange 1980).

Despite some inconsistency between the studies cited above, all of these immunologic studies illustrate two important considerations. First, asbestos exposure is clearly associated with indices of autoimmune responses. Second, although the details vary, the auto-immune responses appear to correlate with asbestos-related disease, suggesting a possible role for the autoimmune responses in the disease process. The temporal relationship among asbestos exposure, autoimmune response, and asbestos-related diseases is uncertain and beyond the scope of the present study. Nevertheless, our findings of strong associations between asbestos and/or vermiculite exposure and risk of SAIDs remained robust after adjusting for objectively characterized pulmonary conditions that could be associated with asbestos exposure.

As described previously, the initial case group was based on those who responded positively to a screening question about autoimmune conditions. Asbestos exposure and SAIDs is a relatively novel avenue of research, so it is not expected that participants would overreport SAIDs because of a suspected association. For the same reason, we would not expect self-reported asbestos exposure to be differentially misclassified with respect to SAID status. As a first step toward improving disease characterization, we conducted a mailing to all participants who indicated having an SAID during the initial screening program. Of those who responded to the mailing, almost 23% were unable to confirm that they had RA, SLE, or SSc. In general, when restricting the analyses to those reporting any physician-diagnosed SAID or RA specifically, the risk estimates for several pathways of asbestos and/or vermiculite exposure were increased. Comparisons of risk estimates between the original group of suspected cases and those who reconfirmed diagnosis should be made with caution, however, because both sets of cases are based on self-report.

Given the unique circumstances of community asbestos exposure and the resulting screening program, it is also possible that there was a case ascertainment error that was biased with respect to exposure. Specifically, those with greater history of asbestos exposure would be more likely to have lung abnormalities or frank asbestos-related disease and would have received more intensive medical care. Better medical care could, in turn, result in a higher likelihood of being diagnosed with other conditions such as SAIDs. This concern is somewhat tempered by the fact that pleural abnormality, the asbestos-related condition that has been most strongly associated with asbestos exposure in this population, was not associated with reporting of SAIDs.

It is also possible, but less likely, that disease status was misclassified among controls. Controls were chosen from among those in the cohort who responded negatively to the screening questions about autoimmune conditions, but we did not confirm the absence of disease among these selected controls. It is possible that some participants with SAIDs did not understand the screening questions and were inappropriately included in the pool of potential controls. This possibility is expected to be of minor concern based on the low prevalence of these conditions in the general population (Jacobson et al. 1997).

This study could suffer from exposure misclassification because these measures were based on self-reported responses as part of a large community-based screening program. Recall bias is a possibility, and persons with chronic health conditions such as SAIDs could overreport past exposure to vermiculite. In the future, we plan to improve on exposure characterization for this cohort, incorporating job exposure matrices and quantitative or semiquantitative assessment of other exposure pathways.

In summary, these preliminary findings provide a unique insight into the risk for autoimmune disease among a population with historical asbestos exposures. These results warrant a more comprehensive case–control study of this population with improved disease and exposure characterization and the incorporation of biomarkers of genetic susceptibility.

## Figures and Tables

**Table 1:** Distribution [*n* (%)] of selected characteristics for participants reporting any SAID, RA, or SLE.

Characteristic	SAIDs^a^ (*n* = 494)	SAIDs^b^ (*n* = 161)	RA (*n* = 129)	SLE (*n* = 30)
Sex				
Male	207 (41.9)	69 (42.9)	61 (47.3)	2 (6.7)
Female	287 (58.1)	92 (57.1)	68 (52.7)	28 (93.3)
Age (years)^c^				
17–44	69 (14.0)	18 (11.2)	15 (11.6)	5 (16.7)
45–64	258 (52.2)	97 (60.3)	80 (62.0)	21 (70.0)
≥ 65	167 (33.8)	46 (28.6)	34 (26.4)	4 (13.3)
Smoking history				
Never	163 (33.1)	51 (31.9)	35 (27.3)	11 (36.7)
Ex-smoker	214 (43.4)	78 (48.8)	64 (50.0)	14 (46.7)
Current	116 (23.5)	31 (19.4)	29 (22.7)	5 (16.7)
Years lived in Libby				
0.5–14	118 (23.9)	37 (22.9)	28 (21.7)	0 (0.0)
15–27	133 (26.9)	41 (25.5)	36 (27.9)	9 (30.0)
28–39	120 (24.3)	47 (29.2)	39 (30.2)	12 (40.0)
≥ 40	123 (24.9)	36 (22.4)	26 (20.2)	9 (30.0)

a Subjects responding positively to original screening question on SAIDs.

b Subjects confirming physician-diagnosed SAIDs in follow-up mailing.

c Age as of 1 January 2000.

**Table 2:** Association between SAIDs and positive findings for chest radiograph and pulmonary function.

Medical evaluation outcome	SAIDs^a^ (*n* = 494)	OR (95% CI)	SAIDs^b^ (*n* = 161)	OR (95% CI)
Pulmonary function test abnormality^c^	90	1.76 (1.34–2.32)	37	2.23 (1.50–3.33)
Pleural abnormality^d^	134	1.15 (0.91–1.45)	46	1.25 (0.87–1.79)
Parenchymal abnormality^d^	27	2.54 (1.51–4.27)	9	2.62 (1.23–5.58)

a Subjects responding positively to original screening question on SAIDs.

b Subjects confirming physician-diagnosed SAIDs in follow-up mailing.

c FVC < 80% predicted and an FEV_1_:FVC ratio ≥ 70% predicted.

d At least two of three B readers observed this type of abnormality on chest radiographs.

**Table 3:** Adjusted ORs^a^ (95% CIs) for vermiculite/asbestos exposure and risk of any SAID or RA specifically, Libby, Montana.

Exposure pathway	SAIDs^b^ (*n* = 494)	SAIDs^c^ (*n* = 161)	RA (*n* = 129)
Potential occupational exposure to vermiculite			
Ever work for W.R. Grace/Zonolite			
Age < 65 years	0.57 (0.29–1.11)	0.74 (0.29–1.91)	0.52 (0.16–1.70)
Age ≥ 65 years	1.47 (0.82–2.63)	2.14 (0.90–5.10)	3.23 (1.31–7.96)
Contract worker for W.R. Grace/Zonolite	1.29 (0.89–1.89)	1.21 (0.67–2.20)	1.29 (0.89–1.89)
Live with W.R. Grace/Zonolite workers	1.19 (0.93–1.53)	1.29 (0.88–1.90)	1.23 (0.80–1.90)
Dust or vermiculite exposure at other jobs	1.33 (1.08–1.64)	1.54 (1.10–2.15)	1.65 (1.14–2.39)
Potential occupational exposure to asbestos			
Worked mixing, cutting, or spraying asbestos	1.39 (0.76–2.54)	0.47 (0.11–2.01)	0.59 (0.14–2.53)
Worked in shipyard/ship construction	1.27 (0.68–2.37)	1.23 (0.46–3.25)	1.80 (0.72–4.46)
Asbestos exposure in the military	1.05 (0.62–1.79)	1.70 (0.84–3.44)	2.11 (1.04–4.30)
Worked in construction-related jobs^d^	1.21 (0.79–1.84)	1.17 (0.60–2.27)	1.32 (0.66–2.65)
Worked as a brake repair person	0.87 (0.49–1.55)	0.65 (0.23–1.86)	0.61 (0.19–2.02)
Activities or experiences with potential exposure			
Frequently handled vermiculite insulation	1.34 (0.99–1.82)	1.60 (1.02–2.52)	1.89 (1.17–3.04)
Used vermiculite for gardening	1.31 (1.06–1.62)	1.70 (1.19–2.43)	1.70 (1.15–2.53)
Frequently recreated near Rainey Creek Road	1.50 (1.18–1.91)	1.67 (1.15–2.43)	1.78 (1.19–2.68)
Frequently played in the vermiculite piles	1.39 (1.02–1.91)	1.85 (1.18–2.92)	2.06 (1.27–3.34)
Frequently played at the ballfield near the plant	1.19 (0.95–1.49)	1.62 (1.14–2.30)	1.74 (1.18–2.55)
Frequently “popped” vermiculite	1.39 (0.96–2.02)	2.11 (1.27–3.52)	1.68 (0.92–3.05)

a Adjusted for restrictive spirometry, parenchymal abnormalities, and smoking history.

b Subjects responding positively to original screening question on SAIDs.

c Subjects confirming physician-diagnosed SAIDs in follow-up mailing.

d Includes carpenter, drywall finisher, insulator, roofer, plumber, electrician, welder, and pipe fitter.

**Table 4:** Crude and adjusted^a^ ORs (95% CIs) risk of any SAID or RA specifically, by number of vermiculite/asbestos exposure pathways.

	SAIDs (*n* = 161)			RA (*n* = 129)		
No. of exposure pathways	Cases/controls	OR	Adjusted OR (95% CI)	Cases/controls	OR	Adjusted OR (95% CI)
0	2/34	1.00 (Referent)	1.00 (Referent)	1/34	1.00 (Referent)	1.00 (Referent)
1	5/101	0.84	0.89 (0.16–4.83)	3/101	1.01	1.02 (0.10–10.23)
2–3	26/345	1.28	1.36 (0.31–6.04)	18/345	1.77	1.79 (0.23–13.93)
4–5	32/364	1.49	1.60 (0.36–7.04)	27/364	2.52	2.51 (0.33–19.18)
6+	96/637	2.56	2.58 (0.60–11.05)	80/637	4.27	3.98 (0.53–29.66)
Trend^b^		*p* < 0.001			*p* < 0.001	

a Adjusted for restrictive spirometry, parenchymal abnormalities, and smoking history.

b Cochran-Armitage trend test.

**Table 5:** Final model adjusted ORs (95% CIs) for vermiculite/asbestos exposure and risk of any SAID or RA specifically, Libby, Montana.

	SAIDs		RA	
Variable	< 65 years (*n* = 115)	≥ 65 years (*n* = 46)	< 65 years (*n* = 95)	≥ 65 years (*n* = 34)
Ever work for W.R. Grace/Zonolite	—a	—	—	3.03 (1.17–7.82)
Dust or vermiculite exposure at other jobs	1.60 (1.07–2.38)	—	1.71 (1.10–2.66)	—
Asbestos exposure in the military	—	2.99 (1.04–8.59)	—	3.31 (1.00–10.96)
Used vermiculite for gardening	1.66 (1.09–2.53)	—	—	3.43 (1.34–8.76)
Frequently played in the vermiculite piles	—	—	1.77 (1.03–3.04)	2.75 (0.75–10.06)
Frequently “popped” vermiculite	—	4.33 (1.59–11.80)	—	—
Restrictive spirometry	3.23 (1.91–5.47)	—	3.09 (1.74–5.47)	2.02 (0.87–4.72)
Parenchymal abnormalities	—	3.29 (1.31–8.25)	—	2.90 (0.95–8.87)
Current or former smoker	—	1.82 (0.89–3.69)	1.75 (1.09–2.82)	—
χ^2^, *p*-value^b^	1.195, 0.75	0.250, 0.62	2.349, 0.80	1.211, 0.88

a Variable did not enter the final model for the given disease/age stratum.

b Hosmer-Lemeshow goodness-of-fit test; small *p*-value suggests that the fitted model is not an adequate model.
